# The Free Hormone Hypothesis: Is Free Serum 25‐Hydroxyvitamin D a Better Marker for Bone Mineral Density in Older Women?

**DOI:** 10.1002/jbm4.10059

**Published:** 2018-06-12

**Authors:** Karl Michaëlsson, Annica Rasmusson, Alicja Wolk, Liisa Byberg, Adam Mitchell, Håkan Melhus

**Affiliations:** ^1^ Department of Surgical Sciences Uppsala University Uppsala Sweden; ^2^ Department of Medical Sciences Uppsala University Uppsala Sweden; ^3^ Institute of Environmental Medicine Karolinska Institutet Stockholm Sweden

**Keywords:** VITAMIN D, EPIDEMIOLOGY, DXA, OSTEOPOROSIS, STATISTICAL METHODS

## Abstract

It is presently unclear whether free serum 25‐hydroxyvitamin D (S‐25(OH)D) better reflects bone health than total S‐25(OH)D. We have previously shown that summer total S‐25(OH)D values are more useful to predict bone mineral density (BMD) than winter values. Our objective was therefore to compare the relative importance of free and total S‐25(OH)D for BMD by season. BMD was measured by dual‐energy X‐ray absorptiometry (DXA) in 5002 Swedish women (mean age 68 years) randomly selected from a large population‐based longitudinal cohort study. Free S‐25(OH)D was analyzed by a commercial ELISA and total S‐25(OH)D by HPLC–tandem mass spectrometry (MS/MS). Free and total S‐25(OH)D co‐varied with season, with 26% and 29% higher values in August compared with those in January–March (nadir). There were no differences in mean BMD between categories of free or total S‐25(OH)D in samples collected during winter. Women with higher total S‐25(OH)D measured during summer had higher BMD at the total hip. Compared with women who had total S‐25(OH)D values above 80 nmol/L during summer, adjusted BMD at the total hip was 6% (95% CI, 1% to 11%) lower for S‐25(OH)D concentrations between 30 and 40 mmol/L, and 11% (95% CI, 3% to 19%) lower for those with total S‐25(OH)D <30 nmol/L. In contrast, free S‐25(OH)D measured during summer was not associated with BMD. Compared with women who had highest free S‐25(OH)D measured during summer (>8.8 pmol/L), those with intermediate (2.4–3.5 pmol/L) and lowest (<2.4 pmol/L) free S‐25(OH)D during summer did not have lower total hip BMD values (3% [95% CI, −2% to 7%] and −2% [95% CI, −8% to 4%]). In addition, we found no added value for the prediction of BMD with the combined measurement of total and free S‐25(OH)D during summer or winter. We conclude that vitamin D status assessed by direct measurements of free S‐25(OH)D does not reflect BMD better than total S‐25(OH)D. © 2018 The Authors. *JBMR Plus* published by Wiley Periodicals, Inc. on behalf of American Society for Bone and Mineral Research.

## Introduction

Less than 0.1% of vitamin D circulates in the free form.^1^ About 85% to 90% of the circulating 25‐hydroxyvitamin D (S‐25(OH)D) is bound to vitamin D binding protein (DBP), and the remaining 10% to 15% to albumin. Free S‐25(OH)D can be indirectly calculated based on measurements of the serum concentrations of total S‐25(OH)D, DBP, albumin, and the affinity between S‐25(OH)D and its binding proteins, or measured directly by equilibrium dialysis, ultrafiltration, or immunoassays.^2^

The “free hormone hypothesis” states that the biological activity of a given hormone is affected by its unbound (free) rather than protein‐bound concentration in the plasma.^3^ This concept has been established for thyroid and sex steroid hormones, and direct measurements of free rather than total thyroid hormones is clinical routine. Similar to thyroid and steroid hormones, vitamin D is hydrophobic and transported in the blood bound to protein carriers. The standard method so far has been to measure total 25OHD. It is presently unclear whether free or bioavailable (free + albumin‐bound) S‐25(OH)D is a better marker of S‐25(OH)D availability and more strongly linked to clinical end points than total S‐25(OH)D.

Chronic vitamin D deficiency is associated with decreased calcium absorption and elevated levels of parathyroid hormone (PTH), which may lead to excessive bone resorption and reduced bone mineral density (BMD).^4^ The main end points investigated in relation to vitamin D status are therefore serum PTH and BMD.^5^ The relatively few studies of free S‐25(OH)D in relation to BMD have used the calculated, not directly measured, free S‐25(OH)D and the results are conflicting.^6^, ^7^, ^8^, ^9^, ^10^, ^11^

We recently reported considerable seasonal variation for the importance of total S‐25(OH)D concentrations on BMD among older women.^12^ Older women with low total S‐25(OH)D concentrations during summer had substantially lower BMD when compared with women who had high concentrations. In contrast, no differences in mean BMD values between categories of total S‐25(OH)D were found during the winter.

To clarify whether free S‐25(OH)D is more strongly linked to BMD than total S‐25(OH)D, we used the same large population‐based cohort of women as in our previous study to investigate the association between directly measured, free S‐25(OH)D and BMD in different seasons contrasted against results for total S‐25(OH)D.

## Materials and Methods

### Study sample

The Swedish Mammography Cohort (SMC) is a population‐based cohort in central Sweden (latitude, 60° N).^13^, ^14^ All women born between 1914 and 1948 living in Uppsala County (*n* = 48,517) and all women born between 1917 and 1948 living in Västmanland County (*n* = 41,786) were asked to respond to a comprehensive food frequency questionnaire (FFQ) when invited to a mammography screening (1987–1990). Completed questionnaires were obtained from 66,651 (74%) and after exclusions, 61,433 women remained in the cohort.^13^, ^14^ In 1997, a second expanded questionnaire was sent to all eligible participants and 38,984 (70%) responded. Between November 2003 and October 2009, we invited a randomly selected subcohort of SMC (SMC Clinical; SMCC) living in the city of Uppsala and surrounding areas, to undergo dual‐energy X‐ray absorptiometry (DXA) measurements (bone, fat, and lean mass), to provide biological samples, and to have height and weight measurements taken. Blood samples were collected in the morning following an overnight fast. The samples were light‐protected and spun in a refrigerated centrifuge, frozen in multiple tubes and stored at −80°C until analysis. A third questionnaire on diet and lifestyle factors was also completed 1 to 3 months before the clinical examination. The participation rate was 65% and the SMCC consists of 5022 women.

### BMD measurements

BMD at the total hip, femoral neck, lumbar spine (L_1_–L_4_), and of the total body were measured using the same DXA (Lunar Prodigy; Lunar Corp., GE Medical Systems, Madison, WI), with use of the same equipment during the study. Osteoporosis was defined as a BMD of 2.5 SD or more below the mean of US white female reference populations aged 20 to 40 years either at the total hip, the femoral neck, or the lumbar spine. The short‐term precision measurement error, based on duplicate measurements with repositioning according to recommendations from the International Society for Clinical Densitometry, varied between 0.8% and 1.5% depending on the measurement site. The long‐term coefficient of variation was <1% for a spine phantom.^15^

### Determination of total 25‐OH vitamin D

The analysis of total S‐25(OH)D, including both 25OHD_2_ and 25OHD_3_, has been described in detail.^(12)^ Briefly, total S‐25(OH)D in serum was determined by high performance liquid chromatography (HPLC)–tandem mass spectrometry (MS/MS) at Vitas, Oslo, Norway (http://www.vitas.no/). Coefficients of variation (CVs) for interassay analyses were between 3% and 6%. Samples from 5002 of 5022 women in the SMCC were analyzed at the same time. The method has a high accuracy (95%) compared with all laboratory trimmed mean (ALTM) spiked samples from the Vitamin D External Quality Assessment (DEQAS) scheme and has been standardized against serum material provided by the US National Institute of Standards and Technology (NIST).^16^

### Determination of free 25‐OH vitamin D

The Free 25OH Vitamin D ELISA (DIAsource, Louvain‐la‐Neuve, Belgium) was used according to the manufacturer's instructions to determine the free S‐25(OH)D concentrations, the sum of free 25(OH)D_2_, and free 25(OH)D_3_. The CV for interassay analyses was about 4%.

### Other measurements

Plasma PTH, calcium, creatinine, cystatin C, albumin, ALAT, beta CrossLaps, and osteocalcin were analyzed using routine methods as described.^12^ Nutrient intakes were estimated by use of data from the FFQs and other lifestyle information was categorized as described.^13^, ^14^

### Statistical analysis

Free and total S‐25(OH)D were plotted by season. We divided our women in six predefined categories of total S‐25(OH)D (<30, 30 to <40, 40 to <50, 50 to <60, 60 to <80, and >80 nmol/L) as in our previous study^12^ and in six correspondingly large categories of free S‐25(OH)D (<2.4, 2.4–3.5, 3.6–4.6, 4.7–6.0, 6.1–8.8, and >8.8 pmol/L). The adjusted percentage differences in BMD values by season (winter as December–February, spring as March–May, summer as June–August, fall as September–November) was estimated (PROC GLM, SAS 9.4; SAS Institute Inc., Cary, NC, USA) were estimated by use of the highest category of the exposure as reference. Moreover, to evaluate the joint influence of lowest total and free S‐25(OH)D on adjusted values of BMD, we combined three categories of total (<30, 30–40, and >40 nmol/L) and three categories of free S‐25(OH)D (lowest five percentiles, percentiles 5–10, highest 90 percentiles) into nine separate categories and used as reference those who had highest concentrations of both total and free S‐25(OH)D. The multivariable model included leisure time physical exercise (less than 1 hour/week, 1 hour/week, 2–3 hours/week, 4–5 hours/week, and more than 5 hours/week), total fat mass (continuous), total lean muscle mass (continuous), body height (continuous), current smoking status, vitamin D supplementation (because use might be related to a general health‐seeking behavior and lower risk of frailty),^17^ bisphosphonate use, ever use of postmenopausal estrogen replacement therapy, previous hip fracture, previous fracture of any type, weighted Charlson comorbidity index, plasma cystatin C, plasma creatinine, plasma ALAT, energy intake, and dietary calcium intake (all continuous). Additional adjustment for educational level (three categories), marital status (living alone), nulliparity, calcium supplementation, menopausal age, and estimated glomerular filtration rate (eGFR) (both continuous) did not affect our estimates more than marginally. Moreover, adjustment by propensity scores^18^ estimated from ordinal logistic regression (PROC logistic; SAS 9.4) including the covariates described in the primary multivariable model revealed similar estimates as after adjustment by individual variables.

Sensitivity analyses were performed by excluding women with an eGFR (based on both plasma creatinine and cystatin C)^19^ below 50 mL/min, ALAT more than three times the upper normal level, women with use of vitamin D supplements, bisphosphonate users, or those who had serum albumin below 35 g/L, calcium above 2.5 mmol/L, and PTH levels higher than 6.9 pmol/L (the upper normal level).

## Results

Characteristics of the women by free vitamin D levels are displayed in Table 1. The mean age of the women was 68 years, with a range of 55 to 86 years. Women with low free S‐25(OH)D levels had on average higher body mass index, although the correlation between free S‐25(OH)D and BMI was moderate (Pearson correlation coefficient; *r* =–0.07, *p* < 0.0001). Correlations were similar for fat mass (*r* =–0.06, *p* < 0.0001) and PTH levels (*r* =–0.07, *p* < 0.0001). Otherwise, there were no major differences in covariates between categories of S‐25(OH)D. Only 0.7% of the women had S‐albumin <35 g/L and 0.4% had a GFR <30 mL/min.

**Table 1:** Characteristics of the Swedish Mammography Cohort Clinical by Free Serum 25‐OH Vitamin D Categories

	Categories of free S‐25(OH)D					
	<2.4 pmol/L (*n* = 244) (4.8%)	2.4–3.5 pmol/L (*n* = 517) (10.4%)	3.6–4.6 pmol/L (*n* = 944) (18.9%)	4.7–6.0 pmol/L (*n* = 1104) (22.1%)	6.1–8.8 pmol/L (*n* = 1625) (32.5%)	>8.8 pmol/L (*n* = 561) (11.2%)
Variable (unit), mean ± SD						
Age (years)	67.8 ± 6.8	67.6 ± 6.9	67.5 ± 6.8	67.6 ± 6.7	67.7 ± 6.6	67.3 ± 6.9
Height (cm)	163.1 ± 6.6	163.6 ± 6.0	163.3 ± 6.3	163.6 ± 6.0	163.6 ± 6.1	164.0 ± 6.0
Body mass index (kg/m^2^)	27.1 ± 4.8	26.4 ± 4.4	26.3 ± 4.4	26.0 ± 4.3	25.6 ± 4.1	25.8 ± 4.0
Total fat mass (g)	28531 ± 8681	27637 ± 8854	27569 ± 9125	26920 ± 8747	26011 ± 8277	26633 ± 8382
Total lean mass (g)	39509 ± 4586	39686 ± 4461	39417 ± 4384	39501 ± 4400	39219 ± 4395	39689 ± 4255
Total bone mass (g)	2315 ± 382	2352 ± 362	2325 ± 382	2328 ± 379	2311 ± 380	2314 ± 375
Total S‐25(OH)D (nmol/L)	45.5 ± 16.2	47.5 ± 15.0	52.0 ± 15.6	58.0 ± 16.5	63.6 ± 17.3	69.0 ± 17.8
Free S‐25(OH)D (pmol/L)	1.62 ± 0.61	3.02 ± 0.33	4.14 ± 0.33	5.33 ± 0.37	7.13 ± 0.80	10.41 ± 1.76
P‐PTH (intact) (pmol/L)	5.5 ± 2.1	5.2 ± 1.8	5.2 ± 1.8	5.0 ± 1.9	5.0 ± 1.9	5.0 ± 1.9
P‐Phosphate (mmol/L)	1.13 ± 0.14	1.17 ± 0.13	1.14 ± 0.14	1.16 ± 0.14	1.16 ± 0.14	1.15 ± 0.13
eGFR (mL/min)	81.7 ± 15.4	81.7 ± 14.9	81.4 ± 15.2	80.8 ± 15.4	81.0 ± 15.2	81.2 ± 14.9
P‐CystatinC (mg/L)	0.94 ± 0.21	0.92 ± 0.20	0.93 ± 0.25	0.94 ± 0.24	0.93 ± 0.23	0.93 ± 0.21
P‐Creatinine (µmol/L)	68.6 ± 12.8	69.2 ± 12.2	69.8 ± 20.2	70.7 ± 14.0	71.0 ± 16.1	70.8 ± 12.9
P‐ALAT (µkat/L)	0.24 ± 0.14	0.24 ± 0.15	0.23 ± 0.14	0.22 ± 0.13	0.23 ± 0.14	0.25 ± 0.16
S‐Albumin (g/L)	42.0 ± 2.5	42.3 ± 3.6	42.5 ± 2.7	42.1 ± 4.3	42.3 ± 2.6	42.0 ± 2.5
P‐Calcium (mmol/L)	2.32 ± 0.11	2.31 ± 0.11	2.31 ± 0.11	2.31 ± 0.12	2.32 ± 0.11	2.29 ± 0.10
S‐CrossLaps (ng/L)	460 ± 179	465 ± 186	461 ± 186	463 ± 191	461 ± 194	458 ± 205
S‐Osteocalcin (µg/L)	25.4 ± 9.8	25.3 ± 9.1	25.0 ± 8.5	24.7 ± 8.5	25.1 ± 9.0	25.1 ± 9.4
Charlson comorbidity index (points)	0.34 ± 0.87	0.20 ± 0.56	0.21 ± 0.58	0.23 ± 0.62	0.20 ± 0.58	0.18 ± 0.58
Age at menopause (years)	49.3 ± 4.4	49.9 ± 3.9	49.9 ± 3.7	50.1 ± 3.4	50.2 ± 3.4	50.0 ± 3.8
Dietary intake						
Energy (kcal/day)	1790 ± 512	1807 ± 587	1757 ± 500	1803 ± 557	1805 ± 544	1780 ± 513
Calcium (mg/day)	1146 ± 469	1132 ± 408	1107 ± 403	1120 ± 391	1114 ± 382	1115 ± 382
Vitamin D (µg/day)	5.7 ± 2.6	5.8 ± 3.1	5.6 ± 2.4	5.9 ± 3.0	5.9 ± 2.5	6.0 ± 2.4
Alcohol (ethanol) (g/day)	5.8 ± 6.9	5.8 ± 6.8	5.8 ± 6.5	6.3 ± 6.7	6.4 ± 7.1	6.8 ± 9.3
Retinol (µg/day)	809 ± 528	851 ± 587	828 ± 750	795 ± 594	807 ± 551	787 ± 558
Phosphorus (mg/day)	1548 ± 524	1587 ± 600	1526 ± 514	1572 ± 544	1572 ± 538	1556 ± 515
Potassium (mg/day)	3380 ± 993	3470 ± 1229	3341 ± 1004	3444 ± 1128	3447 ± 1084	3433 ± 1046
Variable, n (%)						
Any prevalent fracture	52 (21)	83 (16)	157 (17)	212 (19)	292 (18)	96 (17)
Leisure time physical activity						
Less than 1 hour/week	47 (19)	99 (19)	178 (19)	188 (17)	231 (14)	78 (14)
1 hour/week	38 (16)	101 (20)	174 (18)	201 (18)	288 (18)	92 (16)
2–3 hours/week	100 (41)	193 (37)	393 (42)	470 (43)	698 (43)	246 (44)
4–5 hours/week	32 (13)	69 (13)	108 (11)	133 (12)	199 (12)	78 (14)
More than 5 hours/week	26 (11)	55 (11)	88 (9)	108 (10)	201 (12)	64 (11)
Ever use of postmenopausal estrogen	144 (59)	303 (59)	537 (57)	698 (63)	1027 (64)	355 (64)
Living alone	70 (29)	184 (36)	308 (33)	356 (32)	531 (33)	175 (31)
Nulliparity	25 (10)	56 (11)	127 (14)	120 (11)	166 (10)	45 (8)
Education						
<10 years	137 (56)	280 (54)	504 (54)	568 (52)	905 (56)	287 (51)
10–12 years	21 (9)	47 (9)	91 (10)	100 (9)	130 (8)	51 (9)
>12 years	85 (35)	190 (37)	346 (37)	432 (37)	582 (36)	220 (39)
Current smoker	30 (12)	61 (12)	90 (10)	98 (9)	125 (8)	37 (7)
Vitamin D supplement use	32 (13)	34 (7)	84 (9)	98 (9)	174 (11)	67 (12)
Calcium supplement use	18 (7)	22 (4)	74 (8)	88 (8)	164 (10)	63 (11)
Bisphosphonate use	2 (1)	0 (0)	2 (0)	18 (2)	24 (1)	15 (3)

**Figure 1:**
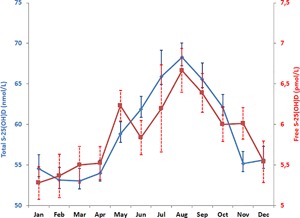
Seasonal variation in free and total serum S‐25(OH)D concentration. S‐25(OH)D = serum 25‐hydroxyvitamin D.

The correlation between free and total S‐25(OH)D was 0.38 (*p* < 0.0001). Average free S‐25(OH)D was 5.9 pmol/L and average total S‐25(OH)D was 58 nmol/L. The values were, however, highly dependent on season of blood draw (Fig. 1). The highest values for both free and total S‐25(OH)D were found in samples taken in August, whereas the lowest were found in January for free and in February to March for total S‐25(OH)D, respectively, with 1.4 pmol/L (26%) and 15 nmol/L (29%) higher values in late summer.

**Figure 2:**
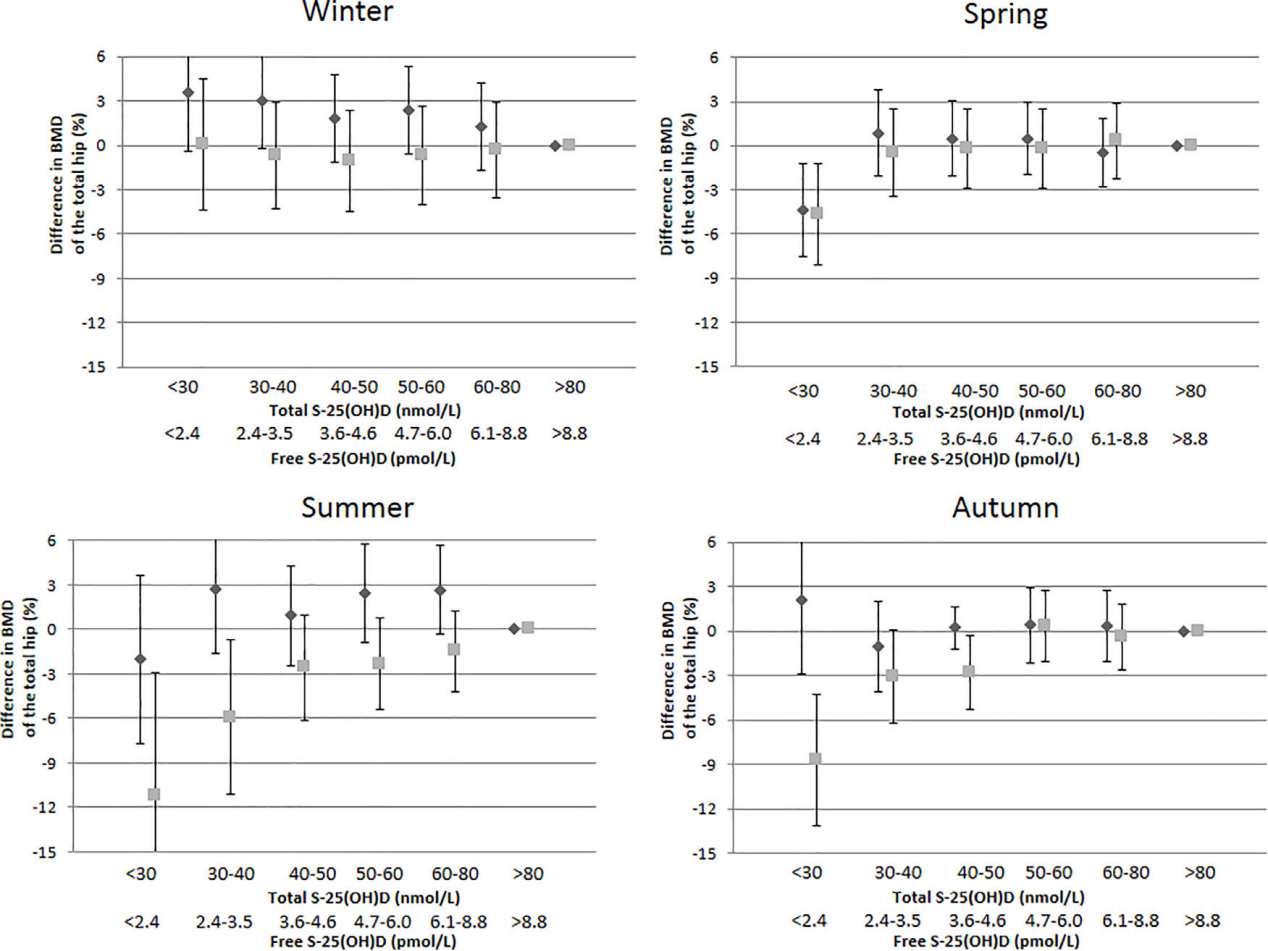
Multivariable adjusted differences in BMD of the total hip within different categories of free (dark gray diamond) and total (light gray square) S‐25(OH)D levels, using >8.8 pmol/L and >80 nmol/L as reference, during the four seasons. The error bars denote 95% confidence intervals. The multivariable model was adjusted for age, leisure time physical exercise, total fat mass, total lean muscle mass, body height, current smoking, bisphosphonate use, ever use of postmenopausal estrogen replacement therapy, weighted Charlson comorbidity index, previous hip fracture, previous fracture of any type, plasma cystatin C, plasma creatinine, plasma ALAT, energy intake, and dietary calcium intake. S‐25(OH)D = serum 25‐hydroxyvitamin D.

Overall, there was a modest positive correlation between summer values of total S‐25(OH)D and total hip BMD; *r* = 0.09 (95% CI, 0.02 to 0.16; *p* = 0.01), but not between free S‐25(OH)D and total hip BMD; *r* =–0.01 (95% CI, −0.08 to 0.06; *p* = 0.76). In contrast to the pattern we have reported previously for total S‐25(OH)D^12^; with a gradually higher BMD of the total hip for total S‐25(OH)D up to 40 nmol/L during summer compared with the reference >80 nmol/L, BMD was not significantly lower than the reference in any of the categories of free S‐25(OH)D during the summer (Fig. 2). Apart from a 4.4% (95% CI, 1.2% to 7.6%) lower BMD in women with free S‐25(OH)D concentrations below 2.4 pmol/L during spring, no seasonal impact on BMD was seen. The results remained essentially unaltered after exclusion of subjects with low eGFR, vitamin D supplement use, bisphosphonate use, and those with low plasma albumin, high plasma calcium, PTH, and ALAT levels, and age below 70 years (data not shown). Results for free summer S‐25(OH)D and total summer S‐25(OH)D were also similar when using femoral neck, lumbar spine (L_1_–L_4_), and total body BMD values as outcomes (data not shown).

**Figure 3:**
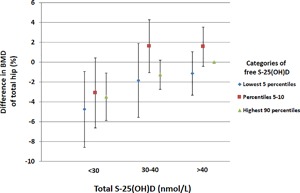
Multivariable adjusted differences in BMD of the total hip for the lowest 5 percentiles (blue diamond), percentiles 5–10 (red square) and highest 90 percentiles (green triangle) of free S‐25(OH)D at total S‐25(OH)D levels of <30, 30–40 and >40 nmol/L, using >40 nmol/L as reference. The error bars denote 95% CIs. The multivariable model was adjusted for age, leisure time physical exercise, total fat mass, total lean muscle mass, body height, current smoking, bisphosphonate use, ever use of postmenopausal estrogen replacement therapy, weighted Charlson comorbidity index, previous hip fracture, previous fracture of any type, plasma cystatin C, plasma creatinine, plasma ALAT, energy intake, and dietary calcium intake. S‐25(OH)D = serum 25‐hydroxyvitamin D.

**Figure 4:**
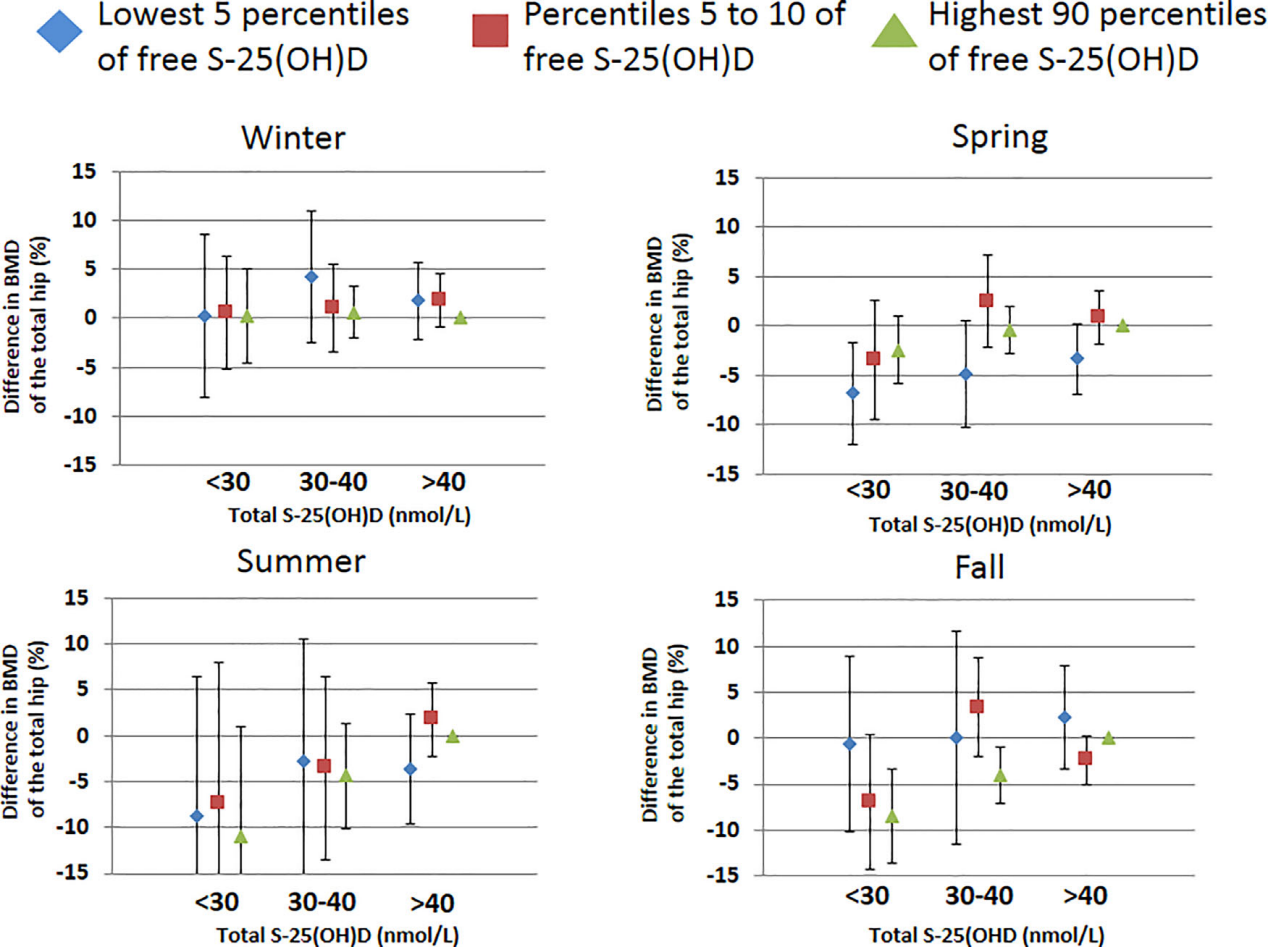
Same as Fig. 3, for each season. Multivariable adjusted differences in BMD of the total hip for the lowest 5 percentiles (blue diamond), percentiles 5–10 (red square) and highest 90 percentiles (green triangle) of free S‐25(OH)D at total S‐25(OH)D levels of <30, 30–40, and >40 nmol/L, using >40 nmol/L as reference. The error bars denote 95% CI. The multivariable model was adjusted for age, leisure time physical exercise, total fat mass, total lean muscle mass, body height, current smoking, bisphosphonate use, ever use of postmenopausal estrogen replacement therapy, weighted Charlson comorbidity index, previous hip fracture, previous fracture of any type, plasma cystatin C, plasma creatinine, plasma ALAT, energy intake, and dietary calcium intake.

To investigate a possible interaction between free and total S‐25(OH)D on BMD, we compared categories of free S‐25(OH)D at different levels of total S‐25(OH)D. As seen in Fig. 3, we found no added value for the prediction of BMD with the combined measurement of total S‐25(OH)D and free S‐25(OH)D. Because summer concentrations of total S‐25(OH)D were the most useful to predict BMD in our previous study, we also analyzed the interaction between free and total S‐25(OH)D by season. As shown in Fig. 4, there were no significant differences in BMD between the lowest five percentiles, percentiles five to 10, and the highest 90 percentiles of free S‐25(OH)D at any level of total S‐25(OH)D during summer. The same was true for winter values, whereas during spring BMD was slightly lower in the lowest five percentiles of free S‐25(OH)D compared to the highest 90 percentiles.

Finally, we wanted to see if free S‐25(OH)D also showed a weaker relation than total S‐25(OH)D with serum PTH. The correlation between free S‐25(OH)D and S‐PTH was *r* =–0.07 [95% CI, −0.04 to −0.10; *p* < 0.0001] and for total S‐25(OH)D *r* =–0.20 [95% CI, −0.17 to −0.23; *p* < 0.0001]. This weaker correlation for free S‐25(OH)D was seen for all four seasons (data not shown).

## Discussion

To our knowledge this is the first study to investigate the relation between directly measured free S‐25(OH)D and BMD. In this large population‐based cohort of Swedish women we previously^12^ showed that season of blood draw is important when interpreting total S‐25(OH)D levels for the determination of cutoff values for vitamin D deficiency and that summer concentrations were the most useful to predict BMD. Because these results at first may seem counterintuitive and the total S‐25(OH)D results are used for comparison in the present study, we will reiterate two important points from that article.^12^: (i) Only 6% of the women failed to reach total S‐25(OH)D levels >40 nmol/L during summer. In general, these individuals will also have low total S‐25(OH)D concentrations during the dark season but the predictive ability of a low winter value will be masked by the higher proportion (21%) of women with total S‐25(OH)D levels <40 nmol/L during winter, ie, those with both low summer and winter values will be difficult to identify using winter values alone. (ii) In that study we also excluded the possibility that the association between summer vitamin D deficiency and low BMD simply represented the selection of a different cohort, by comparing women with low total 25(OH)D concentrations in summer and in winter. We found no indication that those who had low total S‐25(OH)D concentrations in the summer were especially frail individuals with derangements in biomarkers other than those belonging to the vitamin D axis.

In the present study our aim was to investigate whether free, directly measured S‐25(OH)D provides an improved index of vitamin D status in terms of bone health compared to total S‐25(OH)D. Free and total S‐25(OH)D varied similarly over the year, but the correlation of free S‐25(OH)D with BMD was weaker than that of total S‐25(OH)D. There was no added value for the prediction of BMD with the combined measurement of free and total S‐25(OH)D during summer. Thus, measurement of free S‐25(OH)D does not seem to provide more relevant diagnostic information than total S‐25(OH)D in our studied population.

That free serum 25‐hydroxyvitamin D (S‐25(OH)D) did not reflect bone health better than total S‐25‐OHD, means that our results do not support the “free hormone hypothesis” for vitamin D. This hypothesis was first proposed by Recant and Riggs^20^ in 1952. They noted that their patients with nephrotic syndrome were euthyroid despite having low serum concentrations of thyroid hormone and postulated that a then‐undetectable unbound fraction of thyroid hormone in plasma, rather than the protein‐bound fraction, affected biological activity. This fundamental concept has been well established for thyroid hormones. However, its validity for vitamin D has been questioned. S‐25(OH)D, in complex with DBP, is filtered through the glomerulus and reabsorbed in the proximal tubulus by the endocytic receptor megalin, and this endocytosis is required to preserve S‐25(OH)D so that it can be delivered to the cells as the precursor for generation of the active form 1,25(OH)_2_D.^21^ A second cell surface receptor for DBP, cubilin, and a cytoplasmic adaptor protein, disabled‐2, work in conjunction with megalin to facilitate the renal processing of DBP.^22^ Moreover, according to the “free hormone hypothesis,” lack of the carriers should result in increased concentrations of the biologically active free hormone and, consequently, in a condition of aggravated steroid signaling. However, DBP knockout mice are protected against rather than sensitized to vitamin D toxicity, a finding incompatible with the “free hormone hypothesis.”^23^, ^24^

We are aware of only a few studies that have investigated the relation between free S‐25(OH)D and BMD. Importantly, in these studies calculated instead of directly measured free S‐25(OH)D was used, and the results are conflicting. Powe and colleagues^6^ and Johnsen and colleagues^7^ found a better correlation of free and bioavailable S‐25(OH)D than total S‐25(OH)D with BMD, whereas Jemielita and colleagues^8^ did not. In the study by Powe and colleagues,^6^ DBP was measured by monoclonal antibodies, which have been found to be flawed.^25^ In the study by Johnsen and colleagues,^7^ DBP was analyzed by an in‐house radioimmunoassay in a highly selected group of 265 postmenopausal women with reduced bone density. Category analyses of vitamin D status were not used, and the linear association between free S‐25(OH)D and BMD was almost exclusively found in a subgroup not taking vitamin D or calcium supplements. Moreover, the results were not consistent between BMD measurement sites. Jemielita and colleagues^8^ compared monoclonal and polyclonal DBP antibodies. Consistent with the study by Powe and colleagues,^6^ an association between bioavailable S‐25(OH)D and BMD was found in blacks when the monoclonal assay was used. However, no such association was seen when measures of DBP were obtained using polyclonal antibodies.

Our finding of a weaker relation of directly measured free S‐25(OH)D than total S‐25(OH)D with S‐PTH is in agreement with a recent study of healthy premenopausal Spanish women where free and bioavailable S‐25(OH)D levels were estimated by DBP and albumin determinations, and free S‐25(OH)D also measured directly by the same ELISA that we have used. The authors’ conclusion was that determination of free S‐25(OH)D does not offer additional advantages over total S‐25(OH)D for evaluating vitamin D deficiency.^26^ The same conclusion was drawn^27^ in a study of the response of calcium absorption, PTH, and markers of bone turnover to vitamin D. A slightly weaker correlation between S‐PTH and measured free compared to total S‐25(OH)D was also observed in a Norwegian study of prediabetic men and women.^28^ By contrast, two smaller studies suggested that free S‐25(OH)D may be superior to total S‐25(OH)D as a marker of vitamin D bioactivity: Shieh and colleagues^29^ reported that percentage change in PTH was significantly associated with change in measured free, but not total, S‐25(OH)D in 38 participants previously enrolled in a D_2_ versus D_3_ trial, and Schwartz and colleagues^30^ found that free, but not total S‐25(OH)D, was inversely related to S‐PTH in 72 elderly nursing home residents.

It has not yet been established whether free S‐25(OH)D is more informative than total S‐25(OH)D in African Americans. Directly measured free S‐25(OH)D was comparable in Americans of African descent and Americans of European descent despite a higher BMD and lower total S‐25(OH)D in the former, suggesting that African Americans may be incorrectly considered vitamin D deficient if only total S‐25(OH)D is measured.^9^ These results, however, could not be reproduced in an analysis of samples from two clinical trials^31^; directly measured free S‐25(OH)D were lower in black than in white Americans in direct proportion to total S‐25(OH)D.

Whether free S‐25(OH)D is a better measurement of vitamin D status in patients with markedly altered levels of DBP or albumin, such as chronic kidney disease (CKD) or liver failure, remains unclear. Aggarwal and colleagues^11^ found that BMD was positively correlated with bioavailable but not total S‐25(OH)D in adult patients with nephrotic syndrome. In contrast, Denburg and colleagues^(10)^ reported that the correlations of total, free, and bioavailable S‐25(OH)D with cortical volumetric BMD did not differ in childhood CKD. The results of both these studies are difficult to interpret because DBP was determined by the R&D Systems monoclonal assay. Clinical studies comparing total and measured free S‐25(OH)D in CKD populations are currently in progress and should clarify this issue. Patients with liver cirrhosis with synthetic dysfunction as evidenced by low serum albumin (<35 g/L) have lower levels of DBP, total and directly measured free S‐25(OH)D compared to patients with normal albumin. In these patients, however, free S‐25(OH)D was not a better measurement of vitamin D status than total S‐25(OH)D since both free and total S‐25(OH)D displayed the expected relationship with PTH only among those with normal serum albumin levels.^32^ In our population‐based cohort of white women, kidney and liver failure was rare; 0.4% had eGFR <30 mL/min and 0.7% had plasma albumin <35 g/L, and our estimates remained essentially the same after exclusion of subjects with low eGFR, low plasma albumin, and high ALAT levels. Similarly, adjustment for measures of kidney and liver function in the analyses had very little effect on our estimates.

Strengths of our study include the size of the cohort, the population‐based design, a clinically relevant outcome, the direct measurement of free S‐25(OH)D, determination of total S‐25(OH)D using the gold standard, and a large number of conceivable covariates. Because of the unique personal identification number of all Swedish residents, in combination with national healthcare registers, we are capable of a complete comorbidity ascertainment. Our results can be applied to Swedish middle‐aged and elderly women, a population with a high incidence of osteoporotic fractures.

The limitations of our study should be considered when interpreting the results. One limitation common to cross‐sectional observational studies is that they may preclude conclusions regarding causality. Moreover, our results may also not apply to those of different ethnic origins or to men. Our estimates were adjusted for several important covariates, but residual confounding still remains a possible limitation.

We conclude that in a general population, directly measured free S‐25(OH)D is not a better predictor of the clinical outcomes BMD and S‐PTH than total S‐25(OH)D. Our results do not support the notion that the “free hormone hypothesis” is valid for vitamin D.

## Disclosures

All authors state that they have no conflicts of interest.
